# Spread of Influenza Viruses in Poland and Neighboring Countries in Seasonal Terms

**DOI:** 10.3390/pathogens10030316

**Published:** 2021-03-08

**Authors:** Karol Szymański, Ewelina Hallmann, Katarzyna Łuniewska, Katarzyna Kondratiuk, Anna Poznańska, Lidia B. Brydak

**Affiliations:** 1 National Influenza Center, Department of Influenza Research, National Institute of Public Health—National Institute of Hygiene, 00-791 Warsaw, Poland; ehallmann@pzh.gov.pl (E.H.); kluniewska@pzh.gov.pl (K.Ł.); kkondratiuk@pzh.gov.pl (K.K.); lbrydak@pzh.gov.pl (L.B.B.); 2 Department of Population Health Monitoring and Analysis, National Institute of Public Health—National Institute of Hygiene, 00-791 Warsaw, Poland; paula@pzh.gov.pl

**Keywords:** influenza, borders, spread, epidemic, pandemic

## Abstract

In Poland, flu supervision is coordinated by the National Influenza Center at the National Institute of Public Health—National Institute of Hygiene. In this publication, we want to determine geographical trends in influenza virus circulation in the region. A detailed analysis of virological and epidemiological data showed the course of the epidemic season in Poland, as well as in neighboring countries. The spatial differentiation of the incidence of infection between voivodships was examined, as well as compared to countries that border a given voivodship. The results show a significant variation in the incidence of infection in terms of time and space. This points to the need to increase the number of tests and to raise awareness among health care professionals and the public about the probability of an influenza pandemic, as undetected viruses can spread further into the European Union.

## 1. Introduction

The first pandemic of the 20th century in 1918–1919 not only took many lives, currently estimated at around 50–100 million, but also led to huge financial costs [1]. With the above data in mind, the World Health Organization in 1947, at the 4th International Congress of Microbiologists in Copenhagen, proposed the development of a global influenza surveillance system, both epidemiological and virological [2]. The current name of this network is WHO Global Influenza Surveillance and Response System (GISRS) [3]. GISRS functions in the six WHO Collaborating Centers for Influenza Reference and Research. In addition, the WHO is currently working with 149 National Centers for Influenza, including one located at the Influenza Research Department of the National Institute of Public Health—National Institute of Hygiene. Since the epidemic season of 2004/2005, virological and epidemiological supervision of the SENTINEL influenza has also been carried out in Poland [2,4]. Sixteen Provincial Sanitary and Epidemiological Stations (VSES) participate in the supervision carried out in Poland by both GISRS and SENTINEL. These stations supervise the County Sanitary and Epidemiological Stations (CSES) located in each voivodship. The work of these units to monitor influenza virus infections is coordinated by the Influenza Virus Research Institute, National Influenza Center.

The Department of Influenza Research, the National Influenza Center records suspected cases of influenza and influenza-like viruses throughout the epidemic season, i.e., from October until the end of September of the following year. For many epidemic seasons, the peak of the incidence in Poland falls between January and March of the following year [2].

In the Northern Hemisphere, and therefore also in Europe, influenza is most common between November and April each year. The virus is responsible for between 4 and 50 million cases and 15,000–70,000 deaths in Europe related to influenza infection each season [5].

Influenza in humans is an infectious respiratory disease. There are four types of influenza viruses: A, B, C, and D, of which A and B are most common, and type A is responsible for pandemics and seasonal epidemics. Influenza virus belongs to the Orthomyxoviridae family, and has a diameter of 80–120 nm and mass about 170–200 × 10^6^ Da [6]. Genetic material of influenza A and B viruses has eight linear segments in single-stranded RNA, enclosed in a lipid-protein envelope [7,8].

Flu virus infection is characterized by a sudden onset of a fever above 38 °C, dry cough, headache, muscle and joint pain, severe weakness, dry throat, as well as bothersome dry cough that can last about two weeks. In children, diarrhea and vomiting may be an additional symptom of infection. It may take about two days from the moment of infection to the onset of symptoms, which is the incubation phase [2,9,10]. However, studies show that during a typical epidemic season, about 75% of infections are asymptomatic [11].

Influenza viruses can easily spread from person to person. Due to its small dimensions, a virus can be transferred in three different ways: droplet, aerosol, and by contact. Particles of aerosols can remain airborne from minutes to hours, while larger droplets can settle within 2 to 3 m from an infected person. Additionally, influenza virus can remain infectious on non-porous surfaces up to 48 h [6].

That is why a fast and efficient diagnosis of influenza virus infection is so important. The material for research is a swab from the nose and throat, as well as bronchial washing fluids. It is important that the material is collected as soon as possible after the onset of symptoms [5]. Detection methods include rapid tests for influenza virus antigens. These antigens are combined with labeled antibodies in a ready-made buffer, then the complex is attached to the antibodies bound to the nitrocellulose strip in a test cassette, resulting in a colored strip. These methods should be regarded as screening tests and must be confirmed by molecular biology methods. For many years, molecular biology reactions, mainly RT-PCR, have been considered the main standard in many laboratories [12,13].

Currently, there are two types of influenza vaccines used. First is inactivated influenza vaccine (IIV), which is approved for use in persons over six months old. This type of vaccine contains inactivated viruses, which cannot cause influenza infection. A vaccine is administrated intramuscularly in one dose, only for children between six months and eight years if this is their first vaccination for influenza; two doses are required, four weeks apart. Second type is live attenuated influenza vaccine (LAIV), approved for use in persons between 2 to 49 years of age without any underlying medical conditions. This vaccine contains wakened or attenuated influenza virus [14].

Due to the variability of the influenza virus, the WHO updates the composition of the vaccine for both hemispheres every season. National Influenza Centers twice a year sent virus isolates to one of the five WHO Collaborating Centers for Reference and Research of Influenza. Then, the WHO committee, after review of the results of laboratory studies and surveillance, recommends the composition of influenza vaccine for the next epidemic season [15]. Advisory Committee on Immunization Practices (ACIP) recommends that especially high-risk people should get the flu vaccine as soon as the vaccine is available from the pharmacy. Be aware that you can get vaccinated during the ongoing epidemic season, when the virus is already circulating within the population, in which case contact with infected persons should be avoided for seven days [16,17].

The aim of this study is to determine the circulation of influenza viruses in the region in three different seasons in geographical perspective. We also want to illustrate progress in the time of influenza seasons in Poland and surrounding countries.

This part of eastern Europe is populated by approximately 191 million of people, where Poland alone has almost 39 million citizens. Gathering information about influenza virus circulation in this big part of region may be helpful in future healthcare planning.

## 2. Results

**Figure 1:**
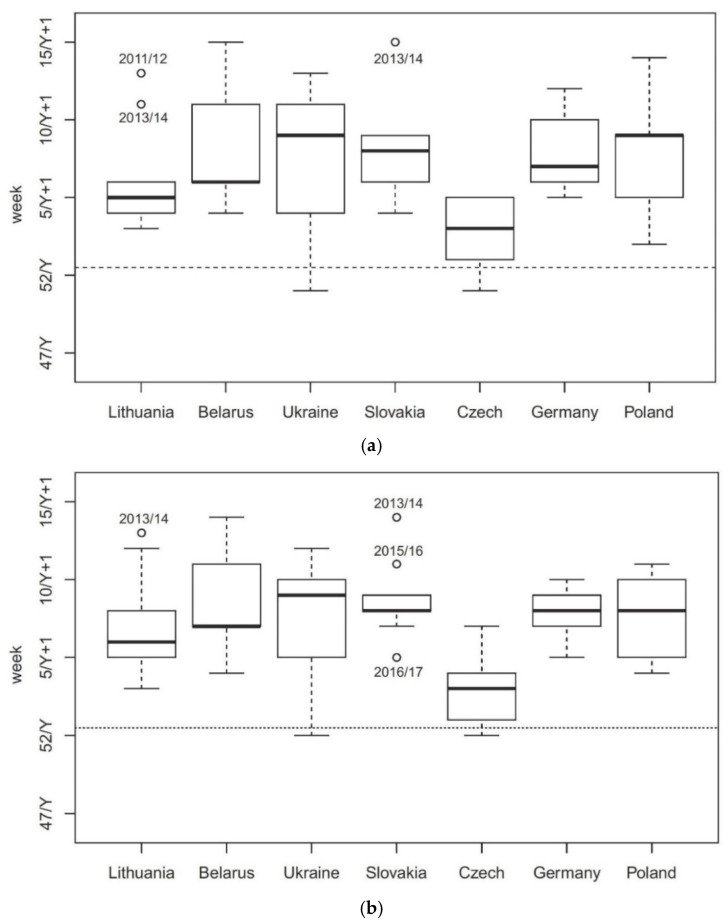
(**a**) Average week of illness and week with the highest number of cases in the epidemic seasons from 2010/2011 to 2018/2019. (**b**) Average week of illness and week with the highest number of cases in the epidemic seasons from 2010/2011 to 2018/2019.

In the seven analyzed countries, the average time of onset in the seasons 2010/2011 to 2018/2019 was the earliest in the Czech Republic (the median of nine seasons is the 16th week of the Y/Y + 1 flu season, i.e., the 3rd week of the Y + 1 year), clearly earlier than in the other countries (where it fell between week 19 and week 22 of the season, that is, between week 6 and 9 of the season Y + 1) (Figure 1a). The difference between the countries is statistically significant (*p* = 0.004). The results of the post hoc tests indicate that the Czech Republic differs significantly from Slovakia (*p* < 0.001), Belarus (*p* = 0.004), Germany (*p* = 0.009), and Poland (*p* = 0.039). There are no statistically significant differences between the other countries.

Very similar results were obtained in the analysis of dominants (Figure 1b). In the Czech Republic, the largest number of cases occurred on average in the third week of the Y + 1 season; in the other countries, between the 5th and 9th week. There are statistically significant differences between the analyzed countries (*p* = 0.001), by pairs between the Czech Republic and Slovakia (*p* < 0.001), Belarus (*p* < 0.001), Germany (*p* < 0.001), and Poland (*p* = 0.001), as well as between Lithuania and Slovakia (*p* = 0.037).

In the epidemic season of 2018/2019 in Poland, 5229 samples were tested for respiratory virus infections under the influenza surveillance system. There were 1818 positive samples registered (which constitutes 34.8% of the tested samples), of which type A infection was confirmed in 1801 patients, and type B in 17 patients. That season, the dominant subtype turned out to be A/H1N1/pdm09, the presence of which was confirmed in 1279 people (70% of positive tests), the A/H3N2/subtype was found in 37 cases, while 485 samples did not have a specific subtype.

**Figure 2:**
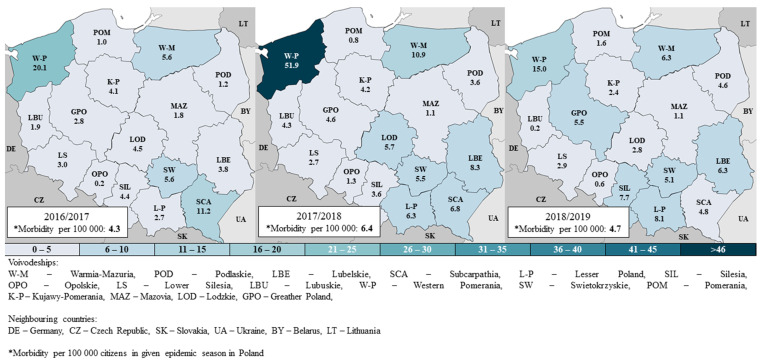
Cases per 100,000 inhabitants in the epidemic seasons of 2016/2017, 2017/2018, and 2018/2019.

The prevalence of positive tests in Poland in the 2016/2017, 2017/2018, and 2018/2019 seasons was 4.3, 6.4, 4.7 per 100 thousand residents, respectively. In all the seasons, the differences between voivodships were observed (Figure 2). The highest rate is found in the Zachodniopomorskie (W-P) voivodship, respectively, 4.7, 8.1, and 3.2 times more than the national average. There are also voivodships (Pomorskie [POM], Opolskie [OPO], Mazowieckie [MAZ]), where the prevalence of influenza is usually very low—less than 2 cases per 100,000 residents. This effect is mainly due to the differences in the number of samples tested—in the 2016/2017, 2017/2018, and 2018/2019 seasons across the country, it was, respectively, 10.0, 15.0, and 13.6 per 100 thousand population; for comparison, in Zachodniopomorskie, it was 55.3, 139.7, and 64.4 (i.e., 5.3, 9.3, and 4.7 times more), while in the Pomorskie voivodship, 1.9, 1.5, 2.1 (i.e., 5.4, 9.7, 6.6 times less than in the country). The percentage of positive samples among the tests carried out throughout the country was 41%, 42%, and 35% in the three consecutive analyzed seasons; clearly lower was only in the Opolskie voivodship: 17%.

Most infections affect people aged 65 or more (in the 2016/2017, 2017/2018, 2018/2019 seasons they constituted 27%, 28%, 32% of confirmed cases) and 45–64 (23%, 23%, 25%, respectively). In these age groups, the most tests were conducted, i.e., 23%, 27%, 31% (65+ group) and 22%, 24%, 27% (45 to 64 years).

**Figure 3:**
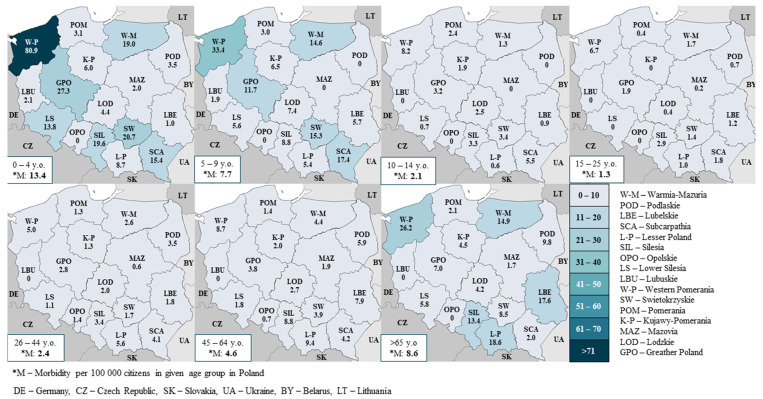
Cases per 100,000 inhabitants in the 2018/2019 season, by voivodships and age groups.

However, the highest incidence rate concerns children aged 0–4 years, in the analyzed seasons 9.8, 16.5, 13.4 per 100 thousand residents, respectively. In this group, the greatest spatial differentiation of the incidence rate is observed—for example, in the 2018/2019 season, from 0 in the Opolskie voivodship to 80.9 in the Zachodniopomorskie voivodship (Figure 3). Relatively small spatial differentiation concerns age groups with lower incidence, i.e., between 10 and 64 years of age.

**Figure 4:**
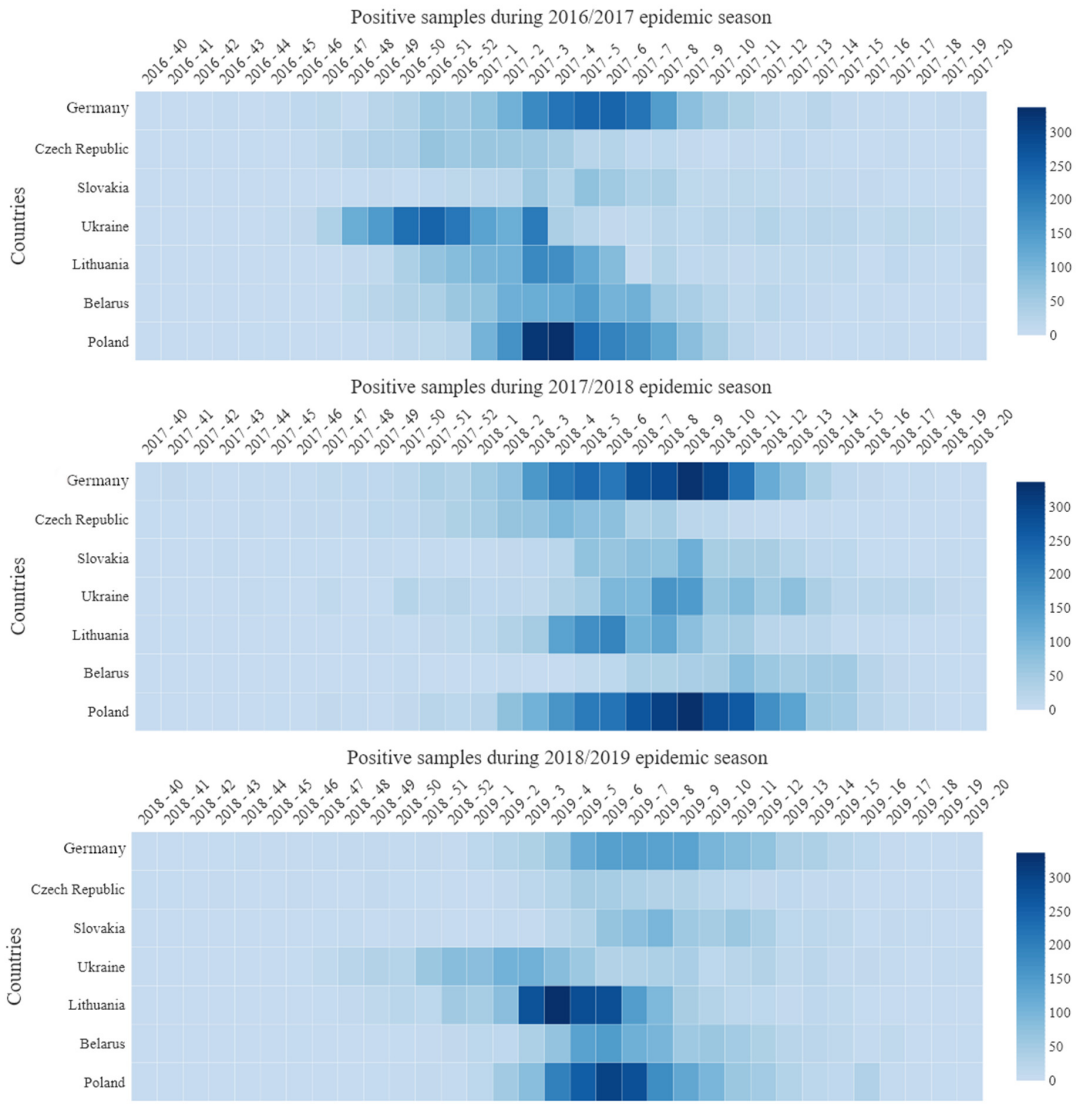
Laboratory confirmations of infection with influenza viruses in Poland and neighboring countries over three epidemic seasons.

Detailed data on the week distribution of influenza cases in three epidemic seasons, i.e., from 2016/2017 to 2018/2019, are presented in the form of a heatmap (Figure 4). It illustrates the general trend and intensity of infection, starting from the 40th week of the calendar year and ending with the 20th week of the following year. The highest values are marked in dark. The statistical analysis of these data is summarized in Table 1. It applies to Poland (and border voivodships) and neighboring countries.

**Figure 5:**
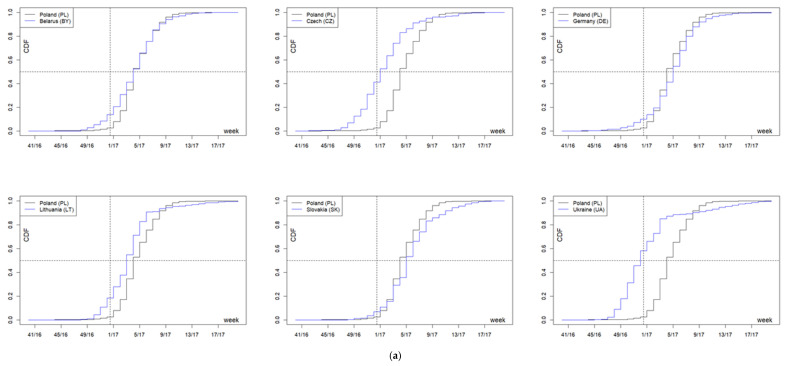

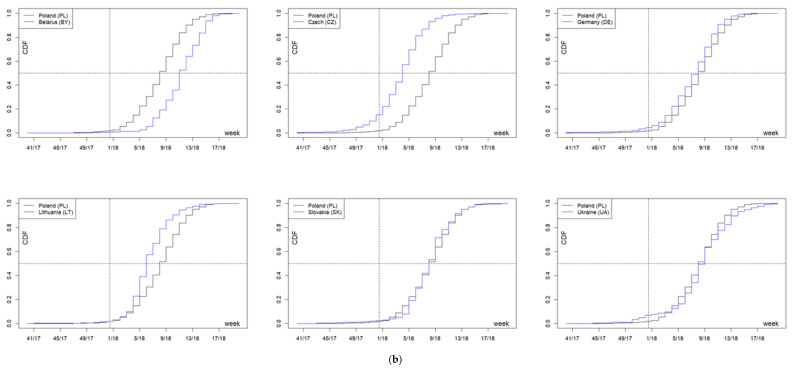

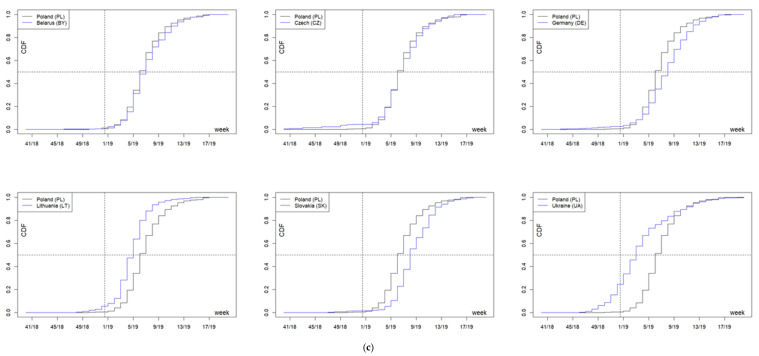
(**a**) Cumulative functions of the distribution of the number of cases over time in the epidemic season of 2016/2017 in Poland in relation to neighboring countries. (**b**) Cumulative functions of the distribution of the number of cases over time in the epidemic season of 2017/2018 in Poland in relation to neighboring countries. (**c**) Cumulative functions of the distribution of the number of cases over time in the epidemic season of 2018/2019 in Poland in relation to neighboring countries.

In the 2016/2017 season, the onset time median in the analyzed countries fell between week 52 of 2016 (52/16, 12 December 2016–1 January 2017) in Ukraine, and week 5 of 2017 (5/17, 30 January–5 February 2017) in Slovakia and Germany. The interquartile range varied from three weeks in Poland to five weeks in Ukraine and the Czech Republic. The distribution of incidence over time in Poland differed in a statistically significant manner from that observed in the other countries (in all cases *p* < 0.001)—cumulative distribution functions (Figure 5a). In Poland, cases of the disease occurred on average in the 4th week of 2017 (4/17, 23–29 January 2017), i.e., later than in Ukraine; in the Czech Republic—in the 1st week of 2017 (1/17, 2–8 January 2017); and in Lithuania—in the 3rd week of 2017 (3/17, 16–22 January 2017), but earlier than in Germany and Slovakia. The difference in distributions between Poland and Belarus is not due to the average onset time (in both countries the 4th week of 2017), but to the fact that the season in Belarus lasted much longer (Figure 5a, Table 1).

There are numerous statistically significant differences observed for onset time between border voivodships and neighboring countries. Thus, in the Warmińsko-Mazurskie voivodship (W-M), the incidence peak appeared a week earlier than in Lithuania; in the Lubelskie (LBE), three weeks later than in Ukraine; in the Podkarpackie (SCA), earlier than in Ukraine and Slovakia; Małopolskie (LP), two weeks earlier than in Slovakia, Śląskie (SIL), two weeks earlier than in Slovakia and two weeks later than in the Czech Republic; Dolnośląskie (LS), two weeks later than in the Czech Republic and a week earlier than in Germany; Lubuskie (LBU) and Zachodniopomorskie (W-P)—respectively, four and two weeks later than in Germany (Table 1).

In the 2017/2018 season, the onset time median in the analyzed countries was between the 4th week of 2018 (4/18, 22–28 January 2018) in the Czech Republic and the 11th week of 2018 (11/18, 12–18 March 2018) in Belarus. The interquartile range varied from three weeks in Lithuania to five weeks in Belarus, Ukraine, Germany, and Poland. The distribution of the incidence over time in Poland did not differ from Slovakia, while it differed statistically significantly from that observed in Lithuania, Belarus, the Czech Republic, and Germany (in all cases *p* < 0.001) and in Ukraine (*p* = 0.021). The cumulative distribution functions are shown in Figure 5b. Influenza in Poland (median: week 8/2018—19–25 February 2018) occurred on average later than in the Czech Republic (week 4/2018—22–28 January 2018), in Lithuania (week 6/2018—5–11 February 2018), and in Germany (week 8/2018—19–25 February 2018), but earlier than in Belarus (week 11/2018—12–18 March 2018), in all cases *p* < 0.001. The comparison between Poland and Germany is interesting—with the same medians (week 8/2018—19–25 February 2018) and a relatively large number of observations in that week (11% each), the share of later cases in both countries was clearly different (41% in Germany and 48% in Poland), which is reflected in the median test. The difference in distributions between Poland and Ukraine is not due to the average time of onset, but to the fact that the season in Ukraine was longer (Figure 5b, Table 1).

Analogous, statistically significant differences are observed for the Polish border regions neighboring Lithuania, the Czech Republic, Germany (on average 1–5 weeks later), and Belarus (3–5 weeks earlier). A similar picture is observed for two voivodships bordering Slovakia—the incidence of the disease in the Podkarpackie (SCA) and Małopolskie (L-P) voivodships was significantly earlier (by three weeks and one week, respectively) compared to both Slovakia and Poland (for distributions and medians in both voivodships, *p* < 0.001; Figure 5b). In turn, infections in the Śląskie (Sil) voivodship were significantly later (on average by one week), comparable only with Slovakia (Figure 5b, Table 1).

In the 2018/2019 season, the onset time median in the analyzed countries was between the 3rd week of 2019 in Ukraine and the 8th week of 2019 in Slovakia and Germany, and the interquartile range was from three weeks in Lithuania to five weeks in Ukraine. The distribution of cases in Poland (median: week 6/2019—4–10 February 2019, interquartile range: four weeks) differed statistically significantly from that observed in Lithuania, Ukraine, Slovakia, and Germany—in all cases, *p* < 0.001 (Figure 5c, Table 1).

Similar differences apply to the border voivodships; the exceptions are the Lubuskie (Lbu) voivodships bordering with Germany (where only two cases were registered—no significant differences) and the Zachodniopomorskie (WP) voivodship, where the onset of the disease was extremely late (median: 9th week 2019), significantly later both in Germany and on average in Poland (*p* < 0.001) (Figure 5c, Table 1).

## 3. Discussion

This work is concerned with the spread of influenza viruses in Poland and neighboring countries over three epidemic seasons—2016/2017, 2017/2018, and 2018/2019. The data indicate a variable viral circulation in the population. There were significant differences in the number of tested and positive samples between individual voivodships in all the epidemic seasons.

The comparison of the analyzed information from the country with the reports of the European Center for Combating and Prevention of Infectious Diseases shows that there are no significant discrepancies in the course of all the epidemic seasons. In the EU countries as well as in Poland and the neighboring countries, the increase, peak, and decrease in the number of cases were recorded in similar periods [5]. In Poland, in all the analyzed seasons, the same virus subtypes circulated as in the other countries.

In Poland, the epidemiological and virological situation in each season varied between voivodships. The differences included the number of patients tested, and thus the differences in the number of positive samples. Regional discrepancies may be related to the ability to order influenza virus testing. The symptoms of infection with the influenza virus may be ignored by patients, which means the patient does not benefit from medical consultations. Research funding is also an important limiting factor. Cheaper methods of detecting the virus, based on rapid tests, which can be considered as screening tests, should be verified using molecular biology methods with higher sensitivity [9].

The analyzed information on the diversification of the number of positive cases, patient reporting, as well as the dependence on the number of tests performed, may indicate that many positive cases are not detected, and therefore they are also not registered. Laboratory testing for infection with the influenza viruses is now the basis of the surveillance system [18].

The low percentage of the vaccinated population, with only 3.9% in the 2018/2019 season, also shows there is insufficient awareness among the population about the risk of infection with the influenza viruses. The highest percentage of the vaccinated population is observed among the elderly. This may be due to the fact that flu vaccinations are reimbursed for this group of patients. These people are much more likely to be infected with the influenza virus due to the weakening of their immune system [10,19]. Doctors are also more likely to order flu virus testing for the elderly—which translates into an increased number of cases per 100,000 inhabitants, which was also reported in this study.

A similar situation is observed among children up to the age of 15. Epidemiological data indicate that a large number of children up to four years of age are most likely to be infected [3]. Their young bodies have not yet fully developed their immune system, which in the case of infection with influenza viruses can lead to a severe course of the disease and numerous complications in various systems, in severe cases leading to hospitalization of the children, mainly under the age of two [6]. It is mainly in children that flu leads to gastrointestinal symptoms such as nausea, vomiting, and diarrhea [2]. Therefore, as in the case of the elderly, this group requires more frequent diagnosis. The results are expressed in the number of cases per 100,000 inhabitants in three age groups: 0–4, 5–9, and 10–14.

People of working age often avoid medical appointments and ignore the symptoms of infection while continuing to work. The obligations of people of this age mean that, in many cases, they do not want to go on sick leave for fear of loss of financial liquidity or the emergence of problems in the workplace. Productive age groups are characterized by very low incidence per 100,000 or incidence. They are also tested less frequently for infection with influenza viruses. However, it is the infections of people of this age that cause the greatest economic damage [2,20]. In Poland, the indirect costs of an influenza epidemic are estimated at approximately PLN 4.3 billion [21].

The analysis of the results showed that the situation in the country does not determine the situation in voivodships. This is indicated by statistically significant data on the examples of voivodships where the disease occurred between two and six weeks earlier or later compared to the general data for Poland. Again, this condition is influenced by significant discrepancies in the number of tests conducted for the influenza viruses.

The statistical analysis of data from Poland compared to information from neighboring countries allows for a broader picture of the epidemic season in the region. This may indicate that the pathogen most frequently comes to Poland from neighboring countries, and that Poland is not initiating an epidemic in the region. Literature data show that, similarly to Poland, the circulation of the influenza virus may significantly differ between seasons also in the Czech Republic [22].

We believe that Poland may be representative for its region, due to high population in relation to neighboring countries. Poland has about 39 million citizens [23], Germany 83 million, Czech Republic 10.6 million, Slovakia 5.5 million, Ukraine 42 million, Belarus 9.5 million, and Lithuania 2.7 million [24]. Furthermore, Poland and neighboring countries share similar percentage of positive samples during different epidemic seasons, and number of tested samples are one of the highest in our region, similar to Belarus and Germany, and higher than Czech Republic, Slovakia, Ukraine, and Lithuania [25].

Poland has a low vaccination rate, 3.3; 3.6; 3.9 percent (2016/2017, 2017/2018, 2018/2019, respectively) [26]. According to the 2018 survey conducted by the Vaccine European New Integrated Collaboration Effort III (VENICE) in collaboration with the European Centre for Disease Prevention and Control (ECDC), similarly low vaccination rates were reported for some of the neighboring countries in the 2016/2017 epidemic season [27].

## 4. Materials and Methods

### 4.1. Statistical Analysis

At the beginning, the disease characteristics were compared over nine epidemic seasons (from 2010/2011 to 2018/2019) in Poland and six neighboring countries (Lithuania, Belarus, Ukraine, Slovakia, the Czech Republic, and Germany). The data came from the SENTINEL system for Poland and FluNet for the neighboring countries. The analyses cover the first 33 weeks of epidemic seasons in each country. The time distribution of incidence in the season in individual countries was described using quartiles, and aggregate results were presented in the form of a box plot. The week with the highest number of cases (the dominant of the distribution) was also determined. The statistical significance of differences between the countries was verified using the Friedman test supplemented with the Conover post hoc test to identify differing pairs.

The incidences in the analyzed regions (countries, Polish voivodships) in the 2016/17, 2017/18, and 2018/19 seasons were analyzed using both the cumulative distribution functions (CDF) and the quartiles of incidence weeks (Table 1). The data were compared using the Kolmogorov–Smirnov test for two empirical distributions (two-sample Kolmogorov–Smirnov test), or the median test, respectively.

A significance level of 0.05 was assumed in all statistical analyses. Due to the multiple comparisons, the statistical significance of the results was corrected using the Sidak correction.

### 4.2. Patients and Samples

The research material consisted of samples collected from patients by both primary health care physicians (POZ) and those collected in hospital during hospitalization. The research was conducted by the laboratories of Provincial Sanitary and Epidemiological Stations, County Sanitary and Epidemiological Stations, hospital laboratories, and the laboratory of the Department of Influenza Research, National Influenza Center at NIPH-NIH. The material used for testing for the presence of influenza viruses included swabs from the nose and throat and bronchial tree washing fluids. The results were analyzed in seven age groups of patients: 0–4, 5–9, 10–14, 15–25, 26–44, 45–64, and >65 years.

### 4.3. Viral RNA Isolation

The first stage of research after receiving the sample is the isolation of the genetic material of the virus. The Department of Influenza Research, National Influenza Center, uses Promega’s Maxwell System for this purpose. The Maxwell 16 Viral Total Nucleic Acid Purification Kit (Promega Corporation, Madison, WI, USA) is used during isolation. Viral RNA was extracted from the samples using 200 µL of material, according to the manufacturer’s instructions.

### 4.4. Typing and Subtyping of Influenza Viruses

Quantitative polymerase chain reaction (qRT-PCR) was used to determine the influenza virus type and the subsequent subtype for positive samples. The Roche Light Cycler 2.0 system was used in the Department of Influenza Research, National Influenza Center. (Roche Diagnostics, Rotkreuz, Switzerland). Primers and probes obtained from the International Reagent Resource run by the Centers for Disease Control and Prevention were used. The reaction was carried out in accordance with the manufacturer’s instructions. RNA was subjected to reverse transcription (50 °C, 30 min). The obtained DNA was subjected to the initial denaturation process (1 cycle at 95 °C for 2 min), followed by 45 cycles of amplification: denaturation at 95 °C for 15 s, annealing at 55 °C for 10 s, and elongation at 72 °C for 20 s. RNA obtained from vaccine viruses proposed for a given season by the World Health Organization (WHO) were used as positive controls:

(1) 2016/2017 Season:
  (a) A/H1N1/pdm09: A/California/7/2009
  (b) A/H3N2/: A/Hong Kong/4801/2014
  (c) Victoria: B/Brisbane/60/2008
  (d) Yamagata: B/Phuket/3073/2013
(2) 2017/2018 Season:
  (a) A/H1N1/pdm09: A/Michigan/45/2015
  (b) A/H3N2/: A/Hong Kong/4801/2014
  (c) Victoria: B/Brisbane/60/2008
  (d) Yamagata: B/Phuket/3073/2013
(3) 2018/2019 Season:
  (a) A/H1N1/pdm09: A/Michigan/45/2015
  (b) A/H3N2/: A/Singapore/INFIMH-16-0019/2016
  (c) Victoria: B/Colorado/06/2017
  (d) Yamagata: B/Phuket/3073/2013

Equipment and research methods used by Provincial Sanitary and Epidemiological Stations.

Each of the Sanitary and Epidemiological Stations conducted research in the field of influenza surveillance in Poland. Below is a complete list of equipment and reagents used in the tests (Table 2).

## 5. Conclusions

Due to the current epidemiological and virological situation in the world and the WHO recommendations, it seems justified to strengthen the GISRS, aimed at capturing the highly pathogenic subtype of the influenza virus. It would be possible, for example, through increased financial expenditure on supervision in Poland, as the vaccination rate in Poland is low. Careful observation of epidemic situation in countries with a low vaccination rate is crucial for surveillance in the region. A low vaccination rate will let influenza virus spread in the community easily. Concluding this, when a virus with pandemic potential shows, lack of immunity will lead to high numbers of infections, hospitalizations, and in worst cases—deaths.

In Poland, during the epidemic season, the peak incidence of influenza usually falls between the 4th and 9th week of the calendar year, i.e., between January and March. Therefore, it would be justified to increase the number of tests to detect influenza virus during this period.

The distribution of the incidence over time in Poland differed in a statistically significant manner from that observed in the other countries. However, types and subtypes of influenza viruses were the same in Poland as in the neighboring countries.

The general epidemiological and virological situation in the country differs from the epidemiological and virological situation in individual voivodships.

Data on the number of cases and laboratory confirmations of people of working age are unrepresentative due to the lack of medical consultations.

Considering it has one of the biggest numbers of population in region and a similar percentage of positive samples as neighboring countries, Poland should be considered as a representative country in the region. As this study shows, based on the statistical analysis that influenza virus migrates to Poland from south-eastern countries, influenza can migrate further to the west, deeper into the European Union.

We believe that similar studies in other countries in the European Union may show different valuable information about virus circulation. This kind of knowledge may help during revision or preparing updated Pandemic Plans in other regions.

## Figures and Tables

**Table 1:** Distribution of cases over time (weeks corresponding to the quartiles of the distribution) in the seasons 2016/17, 1017/18, 2018/19. The calculation covers 33 weeks of each season, starting with the first week.

Country/ Region	2016/17						2017/18						2018/19					
	N	Quartiles			*p* Distr.	*p* Median	N	Quartiles			*p* Distr.	*p* Median	N	Quartiles			*p* Distr.	*p* Median
		1	2	3				1	2	3				1	2	3		
		Week 2017						Week 2018						Week 2019				
	Countries *																	
LT	**881**	**1**	**3**	**5**	**<0.001**	**<0.001**	**926**	**5**	**6**	**8**	**<0.001**	**<0.001**	**1747**	**3**	**5**	**6**	<0.001	<0.001
BY	892	2	**4**	6	<0.001	NS	420	9	**11**	14	<0.001	<0.001	869	5	**7**	9	NS	NS
UA	1417	50/16	**52/16**	3	<0.001	<0.001	924	6	**9**	11	0.021	NS	870	1	**3**	6	<0.001	<0.001
SK	334	3	**5**	8	<0.001	<0.001	534	6	**8**	10	NS	NS	531	7	**8**	11	<0.001	<0.001
CZ	428	51/16	**1**	4	<0.001	<0.001	558	2	**4**	6	<0.001	<0.001	280	5	**7**	9	NS	NS
DE	1522	3	**5**	7	<0.001	<0.001	2403	5	**8**	10	<0.001	<0.001	1182	6	**8**	10	<0.001	<0.001
PL	1537	3	**4**	6	---	---	2408	6	**8**	11	---	---	1771	5	**6**	8	---	---
Polish border provinces **																		
W-M	85	4	**4**	6	LT: <0.001	LT: <0.001	156	8	**9**	10	LT: <0.001	LT: <0.001	91	5	**7**	8	LT: <0.001	LT: <0.001
POD	16	3	**4**	6	LT: NS BY: NS	LT: NS BY: NS	43	7	**8**	9	LT: <0.001 BY: <0.001	LT: <0.001 BY: <0.001	54	5	**6**	8	LT: 0.001 BY: NS	LT: <0.001 BY: NS
LBE	90	1	**4**	5	BY: NS UA: <0.001	BY: NS UA: <0.001	176	4	**6**	11	BY: <0.001 UA: <0.001	BY: <0.001 UA: <0.001	133	4	**8**	10	BY: 0.025 UA: <0.001	BY: NS UA: <0.001
SCA	239	2	**3**	5	UA: <0.001 SK: <0.001	UA: <0.001 SK: <0.001	144	3	**5**	6	UA: <0.001 SK: <0.001	UA: <0.001 SK: <0.001	102	4	**6**	7	UA: <0.001 SK: <0.001	UA: <0.001 SK: <0.001
L-P	95	1	**3**	4	SK: <0.001	SK: <0.001	213	6	**7**	9	SK: 0.001	SK: <0.001	272	4	**6**	8	SK: <0.001	SK: <0.001
SIL	201	3	**3**	4	SK: <0.001 CZ: <0.001	SK: <0.001 CZ: <0.001	166	7	**9**	12	SK: 0.002 CZ: <0.001	SK: 0.004 CZ: <0.001	349	4	**6**	7	SK: <0.001 CZ: <0.001	SK: <0.001 CZ: 0.032
OPO	2	6	**6**	6	CZ: NS	CZ: NS	13	7	**7**	8	CZ: <0.001	CZ: 0.007	6	5	**6**	8	CZ: NS	CZ: NS
LS	83	3	**4**	5	CZ: <0.001 DE: <0.001	CZ: <0.001 DE: <0.001	79	8	**9**	11	CZ: <0.001 DE: 0.001	CZ: <0.001 DE: 0.001	83	6	**7**	8	CZ: 0.034 DE: <0.001	CZ: NS DE: <0.001
LBU	18	6	**9**	10	DE: 0.001	DE: 0.009	44	8	**9**	10	DE: 0.032	DE: 0.014	2	7	**7**	9	DE: NS	DE: NS
W-P	346	5	**7**	8	DE: <0.001	DE: <0.001	884	6	**9**	11	DE: <0.001	DE: <0.001	255	7	**9**	11	DE: <0.001	DE: 0.001

*—statistical significance of differences compared to Poland, **—statistical significance of differences in relation to neighboring countries. W-M—Warmia-Mazuria, POD—Podlaskie, LBE—Lubelskie, SCA—Subcarpathia, L-P—Lesser Poland, SIL—Silesian, OPO—Opolskie, LS—Lower Silesia, LBU—Lubuskie, WP—Western-Pomerania.

**Table 2:** Equipment and reagents kit used in Poland by voivodship sanitary stations.

Equipment	Reagent Kit
LightCycler 96 (Roche)	Real Time Ready Influenza A/H1N1/Detection Set, RealTime Ready RNA Virus Master, LightCycler Multiplex RNA Virus Master, Light Mix Modular EAV RNA Extraction Control (Roche)
LightCycler 480 II (Roche)	Multiplex RNA Virus Master (Roche); probes and starters Modular Dx Kit Inf M2, Modular Dx Kit InfA H3, InfB, Light Mix Kit CC_Hexaplex 480 II; control, IC—Roche RNA Process Control Kit Trial Pack
	FTD Flu (Fast-Track Diagnostics)
	PowerChek Pandemic H1N1/H3N2 Real Time RT-PCR Kit (Kogene Biotech); FTD Flu (Fast-Track Diagnostics)
MX3005 P STRATAGENE	One-tube multiplex PCR for influenza A H1N1, B, H1N1, H3, H5 and H7 (Fast-Track Diagnostics)
CFX96 Bio-Rad	FTD Flu (Fast-Track Diagnostics)
	Allplex Respiratory Panel 1 (Flu/RSV/FluA subtyping) (Seegene)
GeneXpert (Cepheid) 7500 Real-Time PCR (Applied Biosystems)	Xpert Flu A, B, A/H1N1/pdm09
Applied Biosystems 7500 Real-Time PCR System Roche Light Cycler 480 II -RBC Bioscience—MagCore HF 16 Plus	Ribo-prep nucleic acid extraction kit (AmpliSens) MagCore Super/HF 16 Plus Nucleic Acid Extraction Kit (RBC Bioscience) FTD Flu (Fast-Track Diagnostics) FTD Flu Differentiation (Fast-Track Diagnostics)
Rotor-Gene (Qiagen)	PowerChek Pandemic H1N1/H3N2 Real Time RT-PCR Kit (Kogene Biotech)
	PowerChek TM Influenza A/B, Pandemic H1N1/H3N2 Real-Time RT-PCR Kit (Kogene Biotech) FTD Flu (Fast-Track Diagnostic)
No equipment name or manufacturer given	Bosphore H1N1Detection Kitv3 (Anatolia Geneworks)
	Allplex Respiratory Panel 1 (Seegene)

## Data Availability

The data presented in this study are available on request from the corresponding author.
